# Protective effects of Salvianic acid A against multiple-organ ischemia-reperfusion injury: a review

**DOI:** 10.3389/fphar.2023.1297124

**Published:** 2023-11-27

**Authors:** Shiyu Yang, Heming Chen, Wei Su, Yunchun Luo, Jun Liao, Yun Wang, Liyan Xiong, Chuan Zhang, Fei Li, Zhe-Sheng Chen, Tingfang Wang

**Affiliations:** ^1^ School of Medicine, Shanghai University, Shanghai, China; ^2^ Shanghai Eighth People’s Hospital, Shanghai, China; ^3^ Department of Pharmaceutical Sciences, College of Pharmacy and Health Sciences, St. John’s University, New York, NY, United States

**Keywords:** Salvianic acid A, ischemia-reperfusion injury, multiple organs, mechanism of action, protective effects

## Abstract

Ischemia-reperfusion (I/R) injury refers to a new injury caused by reperfusion after the restoration of ischemic tissue or organ blood supply. Salvianic acid A (danshensu) is a primary active ingredient extracted from *Salvia miltiorrhiza*. It has a protective function against I/R injury in the cardiovascular system, brain, liver, kidney, gastrointestinal tract, and other organs. This article reviews evidence of the protective effects of Salvianic acid A and its potential mechanisms of action in organ I/R injury protection. The aim of this review is to investigate the role of Salvianic acid A in the treatment of I/R injury, providing a reference resource that could facilitate subsequent studies.

## Graphical Abstract

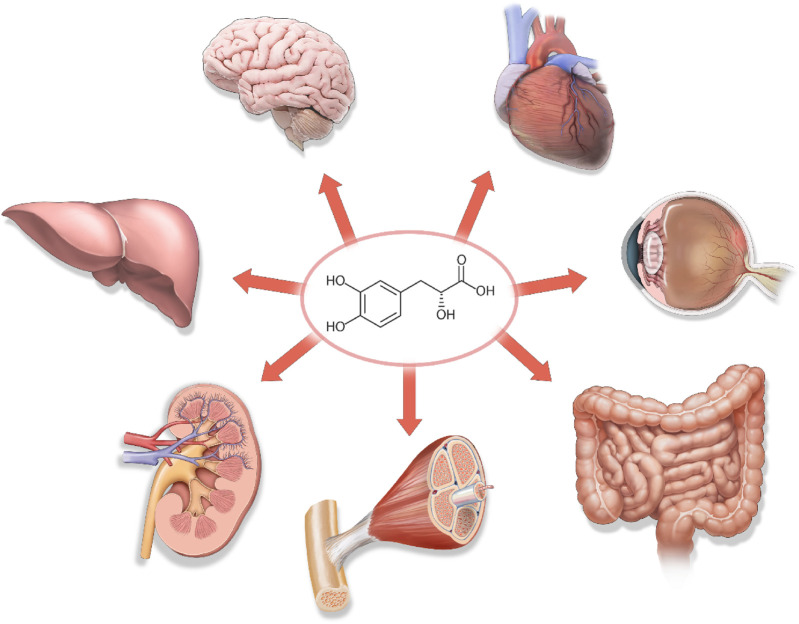

## 1 Introduction

Organ or tissue damage induced by ischemia is the leading cause of fatal diseases, which include myocardial infarction (Heusch and Gersh, 2017), ischemic stroke (Feske, 2021), acute kidney injury (Saat et al., 2016), and organ failure (Jernryd et al., 2020). The timely restoration of blood flow to ischemic tissues using reperfusion therapy is pivotal to the recovery, regeneration, and repair of tissues (Rentrop and Feit, 2015). However, reperfusion can lead to the paradoxical exacerbation of cellular dysfunction, in turn worsening tissue damage; this process is referred to as ischemia-reperfusion (I/R) injury (Wu et al., 2018).

I/R injury is a challenging and life-threatening clinical problem that contributes to the pathology of a wide range of conditions (Anzell et al., 2018). Previous studies have found that the underlying molecular mechanism of I/R injury involves various complex pathological processes, including complement system activation (Gorsuch et al., 2012), platelet–leukocyte aggregation (Al-Mufti et al., 2018), increased free radical production (Baker et al., 1988), calcium overload (Shintani-Ishida et al., 2012), mitochondrial membrane potential destruction (Morciano et al., 2017), reactive oxygen species (ROS) formation (Dambrova et al., 2021), endothelial dysfunction (Bugger and Pfeil, 2020), and endoplasmic reticulum stress (Bai and Lyden, 2015). Severe I/R injury can also cause cell death through the induction of necrosis, apoptosis, autophagy, and other pathways (Kalogeris et al., 2012). Although considerable progress has been made in recent years towards understanding the pathophysiology of I/R injury, safe and effective treatment methods are limited in clinical practice (Bao et al., 2018a). Therefore, it is imperative to explore new drugs to control and treat I/R injury.

Traditional Chinese Medicine (TCM) is thousands of years old and has been used widely to prevent and treat various diseases, particularly cardiovascular diseases (Hao et al., 2017), stroke (Liu et al., 2018), diabetes (Tian et al., 2019), and other chronic diseases (Li et al., 2017). In 2015, Tu Youyou won the Nobel Prize in Physiology or Medicine and delivered a lecture titled “Discovery of Artemisinin: A Gift from Traditional Chinese Medicine to the World,” focusing global attention on TCM (Tu, 2016). *Salvia miltiorrhiza*, the dried root and rhizome of *S. miltiorrhiza* Bunge, is one of the most common and versatile herbs in TCM (Xu et al., 2018). It has been utilized extensively in Asian countries to treat cardiovascular diseases (Bi et al., 2021), hyperlipidemia (Sun G. et al., 2021), atherosclerosis (Cho et al., 2013), and cerebrovascular diseases (Lin and Hsieh, 2010). The main active pharmaceutical ingredients of *S. miltiorrhiza* can be categorized as lipid-soluble tanshinones and water-soluble phenolic acids (Fei et al., 2017). Recently, these phenolic acids have been increasingly attracting attention because they are the chief constituents of aqueous decoctions, which are the most used type of *S. miltiorrhiza* in clinics (MEIm et al., 2019). Specifically, total salvianolic acid, a group of water-soluble phenolic acids isolated from *S. miltiorrhiza*, has been granted approval by the State Food and Drug Administration for the treatment of cardiovascular and cerebrovascular diseases in China (Han et al., 2017).

**FIGURE 1:**
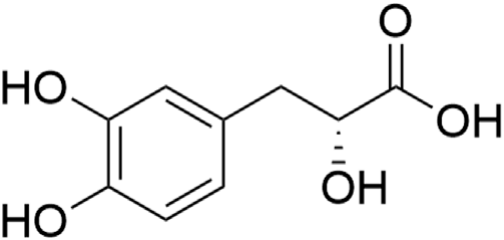
Chemical structure of Salvianic acid A.

Among these water-soluble phenolic acids, Salvianic acid A [also named danshensu (R)-3- (3, 4-dihydroxyphenyl)-2-hydroxypropanoic acid (Figure 1)] is one of the basic constituent structures of various salvianolic acids, and its pharmacological properties and molecular mechanisms have been studied extensively (Bianconi et al., 2018). Extensive experimental evidence has demonstrated that Salvianic acid A has a broad scope of pharmacological effects that protect against myocardial I/R and cerebral ischemia injury (Choi and Pile-Spellman, 2018), improve microcirculation (Jia et al., 2022), protect endothelial cells (Yang et al., 2010), and exert anti-inflammatory (Xu et al., 2021), anti-oxidant (Guo et al., 2013), antiplatelet, antithrombotic, and antithrombotic properties (Dery et al., 2022). As a result, Salvianic acid A has already been used to alleviate angina pectoris and treat coronary heart disease and is currently undergoing phase II clinical trials in China (drug clinical trial registration number: CTR20201177).

**FIGURE 2:**
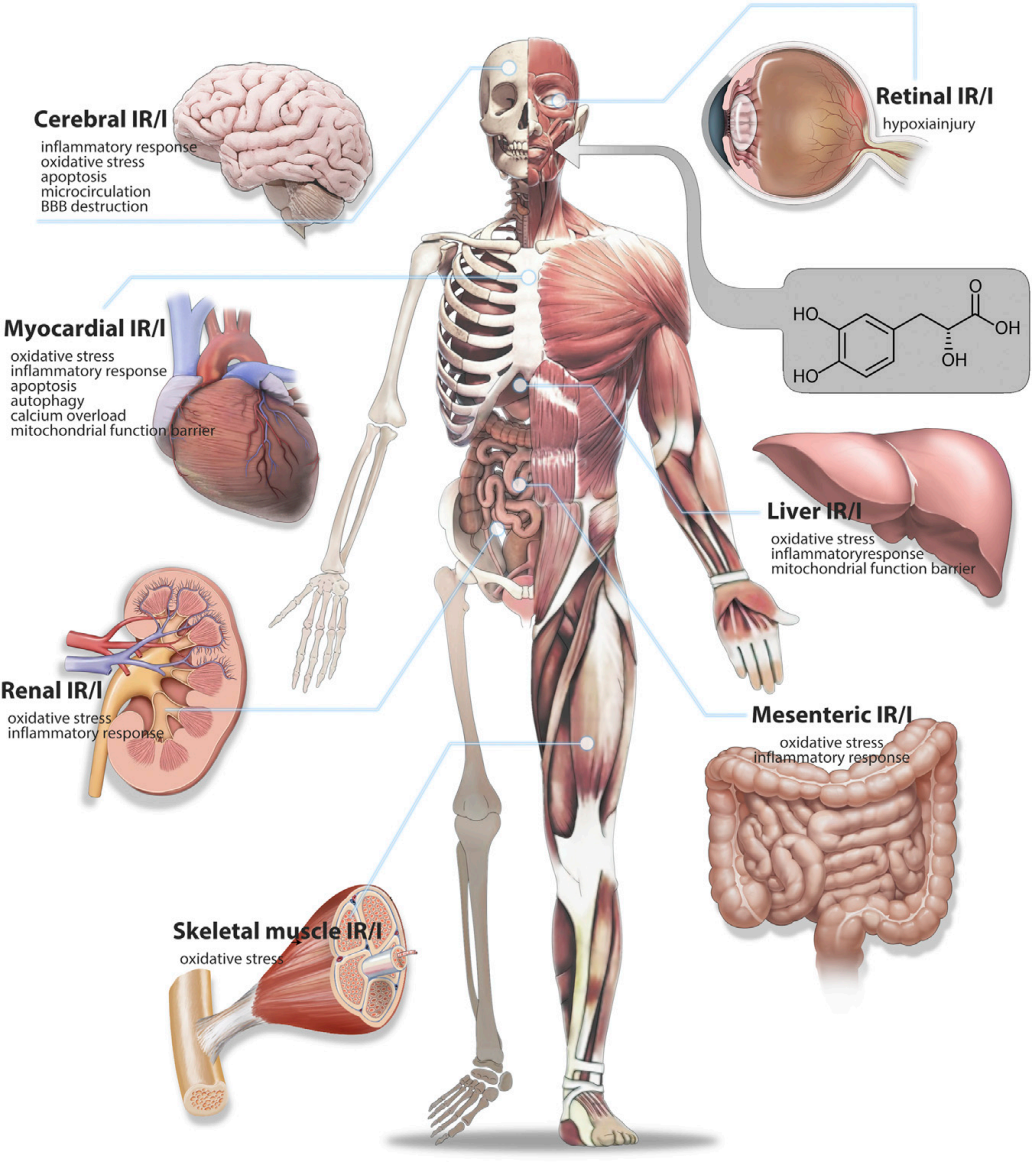
Therapeutic mechanism of Salvianic acid A against ischemia-reperfusion injury in various organs.

Several studies have demonstrated that Salvianic acid A has a strong protective effect on multiple organs, such as the heart, brain, lungs, kidneys, and liver (Bao X. Y. et al., 2018). Despite several published reviews on the cardiovascular pharmacology of Salvianic acid A, to the best of our knowledge, the mechanism of the protective effect of Salvianic acid A against multiorgan I/R injury has not been summarized. In the present review, we summarize research progress on Salvianic acid A in the treatment of multiple-organ I/R injury, aiming to enhance our understanding of the pharmacological mechanisms and provide novel insights for further in-depth research. The specific mechanisms of Salvianic acid A in the prevention of I/R injury of diverse organs are shown in Supplementary Table S1; Figure 2.

## 2 Protective effect of Salvianic acid A against cardiac I/R injury

Myocardial infarction (MI) remains the leading cause of death and morbidity and has become a serious global public health problem (Schirone et al., 2022). Timely and effective restoration of coronary blood flow after MI is a standard therapeutic strategy (Tibaut et al., 2017). Paradoxically, reperfusion itself would result in incremental myocardial damage, which is termed I/R injury (Yellon and Hausenloy, 2007). Research indicates that the mechanism of myocardial I/R injury (MIRI) is complex, with multiple physiological and pathological processes, such as oxidative stress (Guo et al., 2010), inflammatory response (Yao et al., 2022), intracellular Ca^2+^ overload (Shintani-Ishida et al., 2012), mitochondrial dysfunction (Zhou et al., 2021), apoptosis, and autophagy (Xing et al., 2022). Such factors often work synergistically, leading to the onset and progression of MIRI (Hausenloy and Yellon, 2013).

**FIGURE 3:**
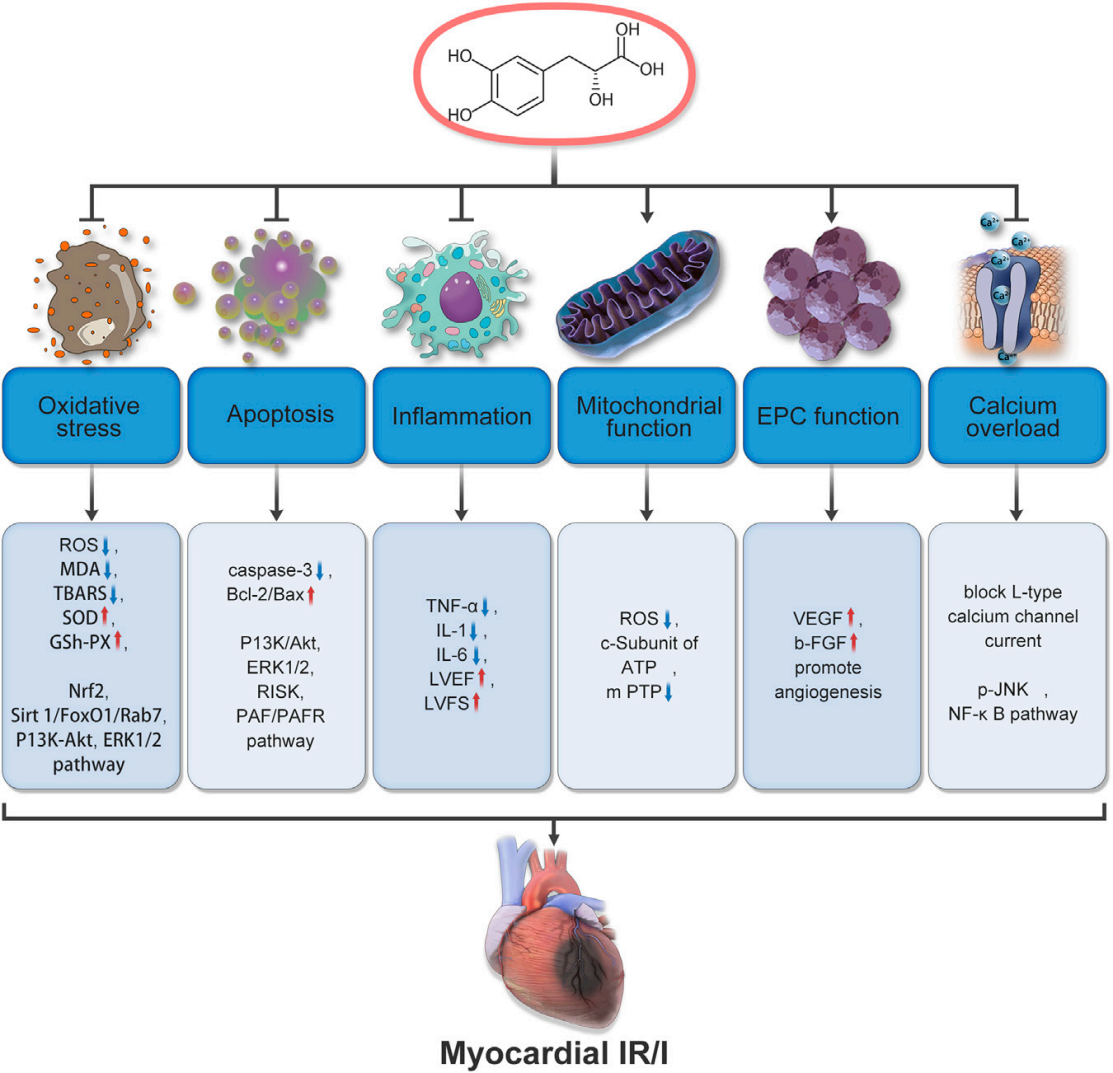
Mechanisms underlying the therapeutic effects of Salvianic acid A in cardiac ischemia-reperfusion injury.

According to reports, the mechanism by which Salvianic acid A prevents and treats MIRI is not only related to coronary artery regulation and anti-platelet effects to improve blood circulation but also involves antioxidant and anti-inflammatory effects, autophagy regulation, anti-apoptosis mechanisms, calcium overload regulation, and endothelial cell protection, wherein many signaling pathways and related proteins play important roles. For example, regulation of the PI3K/AKT signaling pathway results in antioxidant and anti-apoptotic effects; inhibition of the JNK/NF-KB signaling pathway reduces the expression of TRPC6 and plays an anti-calcium overload role (Gao et al., 2017; Gao et al., 2022; Ge et al., 2021) (Figure 3). Furthermore, administration of Salvianic acid A alone or in combination with other Chinese medicinal products has potential preventive and therapeutic effects on MIRI. In the following sections, we briefly elaborate on the mechanism of action of Salvianic acid A in the treatment of MIRI through different pathways.

### 2.1 Anti-oxidative effects

Oxidative stress is defined as the imbalance between oxidative and antioxidative processes *in vivo* (Sies, 2015). The overproduction of ROS and the release of a range of transcription factors that regulate oxidative stress are essential in triggering oxidative responses in ischemic cardiomyocytes (Kurian et al., 2016).

Numerous studies have shown that Salvianic acid A can clear excess ROS (Huang et al., 2019; Ibáñez et al., 2015; Jia et al., 2019a), reduce malondialdehyde (MDA) levels and thiobarbituric acid reactive substances (Kalogeris et al., 2016; Li et al., 2012; Li et al., 2016), and enhance the endogenous antioxidant defense molecules superoxide dismutase (SOD) and glutathione peroxidase (GSH-PX) in a rat MIRI model (Sun D. W. et al., 2019). Therefore, the oxidation of myocardial fatty acids can be inhibited to improve myocardial energy metabolism under pathological conditions and has cardiac protective effects on isolated hearts (Zhang et al., 2010), preventing oxidative stress during I/R injury. The signaling pathways involved include Nrf2 (Li et al., 2018; Pachel et al., 2016; Patel and McMullen, 2017), Sirt1/FoxO1/Rab7 (Sun D. W. et al., 2019), PI3K-Akt, and ERK1/2 (Xu H. et al., 2020). Furthermore, Salvianic acid A reportedly showed antiarrhythmic and cardiac protective effects against hypertrophic heart I/R injury in an I/R model of myocardial hypertrophy by increasing SOD activity and decreasing MDA content (Tang et al., 2011).

Salvianic acid A in combination with other traditional Chinese medicines has also been demonstrated to have a protective effect against myocardial ischemic injury. Studies have shown that paeonol combined with Salvianic acid A can enhance the cardiac antioxidant defense system by regulating the Nrf2/HO-1 and PI3K/Akt pathways and play a cardiac protective role in isoproterenol-induced MI in rats (Qian et al., 2002). The protective effect of salvianolic acid A against MI is mainly reflected in its ability to reduce isoproterenol-induced MI by regulating two signaling pathways against oxidative stress and apoptosis after myocardial cell injury. In another study (Wu et al., 2007), puerarin combined with Salvianic acid A reduced infarct size caused by ischemic injury in a dose-dependent manner through its antioxidant and anti-lipid peroxidation properties and had a significant protective effect against acute ischemic myocardial injury in rats.

### 2.2 Anti-apoptosis

Apoptosis is the process whereby cells “commit suicide” by activating an intracellular death program (Xu et al., 2019). Previous studies have shown that I/R can lead to cardiomyocyte death through a variety of pathways, including apoptosis (Wang et al., 2016). Salvianic acid A acts against apoptosis in injured cardiomyocytes through the activation of the PI3K/Akt, ERK1/2 (Yin et al., 2013), RISK (Guan et al., 2013), and PAF/PAFR pathways and by weakening caspase-3 activity and increasing the Bcl-2:Bax ratio (Wang et al., 2021), thus reducing the release of downstream apoptotic factors, preventing the loss of cardiomyocytes, and minimizing the cardiac damage caused by MIRI.

Of note, the role of Salvianic acid A in alleviating MIRI appears to be linked to its anti-apoptotic pharmacological activity. Hu et al. (2016) first demonstrated that the combination of Salvianic acid A and hydroxyl safflower yellow A (HSYA) can exert antioxidant and anti-apoptotic effects by activating the Akt/Nrf2/HO-1 signaling pathway to prevent myocardial cell damage. Salvianic acid A performs an important role in anti-apoptosis, and HSYA has an essential anti-oxidation role. Guan et al. (2013) compared the activities of several major active components in danhong injection in terms of antioxidant, anti-inflammatory, and anti-apoptosis properties and found that Salvianic acid A had strong anti-apoptotic effects. The results of such studies enhance our understanding of the mechanisms of function of Salvianic acid A in preventing and treating MIRI.

### 2.3 Anti-inflammation

During cardiac ischemia, a large number of pro-inflammatory factors are released, causing the adhesion and migration of neutrophils and vascular endothelial cells, which eventually accumulate around myocardial tissue to release lysosomal enzymes, causing damage to myocardial cells and becoming a key factor inducing cardiac I/R injury (Qiu et al., 2018).

The anti-inflammatory effect of Salvianic acid A on myocardial cells has been widely studied and reported. Salvianic acid A can reduce the levels of tumor necrosis factor alpha (TNF-α), interleukin (IL)-1, and IL-6 (Quan et al., 2012); increase left ventricular ejection fraction levels and left ventricular fractional shortening; and reduce cell swelling, inflammatory cell abundance, and MI area (Jia et al., 2019b). Salvianic acid A may have a protective effect on the myocardium by inhibiting the inflammatory response to MIRI.

### 2.4 Improvement of mitochondrial function

Mitochondria are the most important organelles supplying energy to cardiomyocytes. Damage to mitochondria during MI results in an insufficient energy supply. After reperfusion, mitochondria in ischemic tissue cannot quickly recover their normal form, and ischemic injury persists for a period before improvement occurs, aggravating myocardial cell injury and even causing death (Anzell et al., 2018). The stability of mitochondrial function is closely related to the mitochondrial membrane potential and the mitochondrial permeability transition pore (MPTP) (Griffiths, 2012).

Studies have shown that Salvianic acid A can inhibit opening of the MPTP, stabilize the mitochondrial membrane potential of cardiomyocytes, improve mitochondrial function, and alleviate cardiac I/R injury both *in vivo* and *in vitro* by preventing mass ROS production or inhibiting the expression of the c-subunit of adenosine triphosphate (Ren et al., 2019). However, because the relationship between mitochondria and MIRI is not fully understood, further studies exploring the role of mitochondria in the treatment of MIRI using Salvianic acid A are necessary.

### 2.5 Regulation of autophagy

Autophagy is a key target in the treatment of I/R injury (Aghaei et al., 2019). Excessive autophagy in the myocardial I/R stage leads to the degradation of key proteases in the heart, resulting in increased cell death and aggravated heart injury (Lin et al., 2018). Several studies support the function of Salvianic acid A in the regulation of autophagy-related pathways. Fan et al. (2016) investigated the protective effect of Salvianic acid A on cardiomyocytes and cardiac I/R injury in an isolated rat heart model. Salvianic acid A pretreatment activated the targeted autophagy and apoptosis pathways through mammalian target of rapamycin (mTOR), causing the downregulation of autophagy-related proteins including LC3-II, Beclin-1, and p62, thus alleviating MIRI. Furthermore, Xie et al. (2021) discovered that a novel Salvianic acid A derivative (DT-010) can alleviate oxidative stress-induced autophagy injury in cardiomyocytes via the AMPK–mTOR–Ulk1 signaling pathway and is a potential drug for use in the treatment of MIRI.

### 2.6 Reduction of calcium overload

During MIRI, calcium overload can cause the generation and release of downstream active factors, induce harmful reactions in cells, and interact with inflammation, oxidative stress, and other mechanisms to cause or aggravate myocardial cell injury (Soares et al., 2019).

Salvianic acid A inhibits the influx of calcium ions at the cellular level. Lam et al. (2007) used a 5-HT precontracted rat model and found that Salvianic acid A had a cardioprotective effect on the heart by dilating coronary vasculature through inhibition of Ca^2+^ influx in vascular smooth muscle. At the same time, Salvianic acid A has a regulatory effect on calcium channels. Salvianic acid A can block L-type calcium channel current on myocardial cell membranes (Song et al., 2016). It can also reduce the expression of calcium channel protein TRPC6 by inhibiting p-JNK, nuclear factor kappa-B (NF-κB), and other signaling pathways, ultimately reducing Ca^2+^ influx and the damage caused by calcium overload in myocardial cells (Meng et al., 2016).

### 2.7 Improvement in endothelial progenitor cell function

Endothelial progenitor cells (EPCs) have a self-proliferation and multi-differentiation capacity (Chopra et al., 2018). In the event of tissue ischemia, EPCs in bone marrow can directly form blood vessels, differentiate into endothelial cells, and integrate with ischemic tissue to engage in the generation of new blood vessels (Sun et al., 2021b; Tani, 1990).

Research on the potential relationship between Salvianic acid A and EPCs could yield novel angiogenesis therapy tools. Yin et al. (2017) found that the cardioprotective effect of Salvianic acid A in an MI rat model may be associated with the promotion of myocardial neovascularization. The potential mechanisms include enhancing EPC survival in a hypoxic environment through Akt phosphorylation and accelerating angiogenesis in EPCs through the SDF-1α–CXCR4 axis. The authors also found that Salvianic acid A increased vascular endothelial growth factor (VEGF) and basic fibroblast growth factor expression in myocardial tissue surrounding the infarcted area after MI, significantly promoting new blood vessel growth in the marginal region of the infarcted zone, which can alleviate MIRI and cardiac insufficiency.

Recently, researchers have conducted many studies on the therapeutic aspects of Salvianic acid A in the treatment of MIRI and have found that Salvianic acid A does not act through a single pathway but rather synergistically modulates multiple mechanisms to achieve a protective effect (Sun DW. et al., 2019). However, how the various pathways work synergistically to achieve this effect needs to be studied further. In addition, more research is necessary to investigate the use of Salvianic acid A in combination with other drugs in MIRI treatment.

## 3 Protective effects of Salvianic acid A against cerebral I/R injury

Cerebral I/R injury (CIRI) is one of the most common cerebrovascular diseases. It mainly occurs when blood vessels recanalize after cerebral ischemia and blood reperfusion further aggravates the pathological injury of ischemic tissue, causing secondary injury to patients with ischemic stroke and greater damage to the nervous system (Wang et al., 2022; Xia, 2018). The pathological mechanisms underlying CIRI are complex, involving a combination of calcium overload, excitatory amino acid toxicity, mitochondrial dysfunction, oxygen free-radical accumulation, blood–brain barrier (BBB) destruction, autophagy, and inflammation (Xue et al., 2022).

**FIGURE 4:**
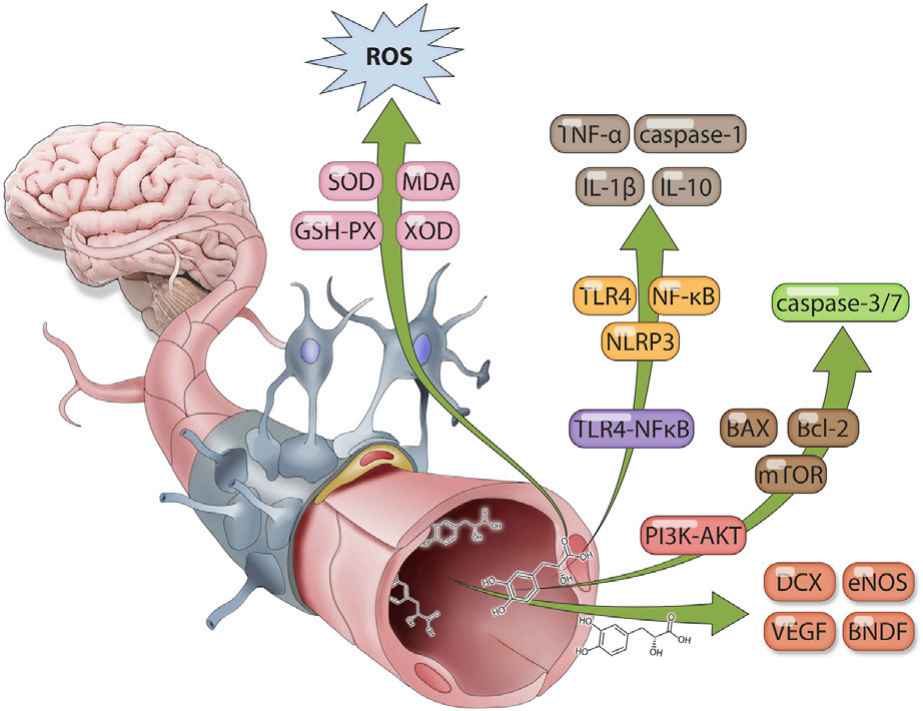
Therapeutic mechanism of Salvianic acid A against cerebral ischemia-reperfusion injury.

Earlier work suggests that Salvianic acid A exhibits a protective effect against cerebral ischemic disease, diminishing cerebral infarct volume and brain tissue edema, promoting neuronal and vascular regeneration, and improving CIRI (Jia et al., 2022). Various mechanisms underlying CIRI treatment have been reported, including anti-oxidative stress, the promotion of anti-inflammation, endogenous neurogenesis, and anti-apoptotic effects. Further research should be carried out to investigate additional protection mechanisms (Yuan et al., 2010) (Figure 4). However, because of low lipid solubility, the ability of Salvianic acid A to penetrate the BBB alone is limited. Therefore, most existing studies use a synthetic Salvianic acid A carrier system or derivatives to improve the BBB penetration rate of Salvianic acid A and increase the effective concentration of the drug to promote the therapeutic effects of the drug (Li et al., 2023) 18.

### 3.1 Anti-oxidative effects

After brain tissue ischemia, high amounts of ROS are produced rapidly and cause organelle damage, and the rapid recovery of blood oxygen supply during reperfusion directly leads to the second explosion of ROS generation, thus leading to brain reperfusion injury (Rodrigo et al., 2013). Salvianic acid A, as an oxygen free-radical scavenger, has an inhibitory effect on the overproduction of oxidation factors during CIRI. Yuan et al. (2010) found that in the cerebral cortex of model rats, Salvianic acid A can improve cerebral I/R injury by raising the activities of SOD and GSH-PX while decreasing xanthine oxidase activity and MDA expression to scavenge excessive ROS, reduce the level of cerebral oxidative stress, and alleviate cerebral infarction. Another study synthesized the hybrid of Tanshinol borneol ester (DBZ) with reference to traditional Chinese medicine theory and concluded that DBZ has antioxidant activity in I/R injury mouse models (Yuan et al., 2018). Specifically, DBZ can suppress lipid peroxidation, raise SOD and GSH-PX activities in model mouse brains, and reduce oxidative stress and MDA levels in the brain.

### 3.2 Promotion of endogenous neurogenesis

Cerebral ischemia can stimulate endogenous neurogenesis in the brain as a defensive response to tissue injury (Koh and Park, 2017). Endogenous neurogenesis contributes to brain tissue repair after ischemic stroke and improves brain neurological function after stroke (Marques et al., 2019). Salvianic acid A can promote cerebral angiogenesis to some extent. Wei et al. (2018) found that in a focal stroke model of mice, chronic Salvianic acid A treatment promoted endogenous neurogenesis and collagen generation by up-regulating the expression of doublecortin-positive cells, VEGF, brain-derived neurotrophic factor, endothelial nitric oxide synthase, and other key nutritional and regenerative factors. This is manifested by increased growth of new neurons, endothelial cell/vascular proliferation, and collateral artery formation, which contribute to the restoration of local blood flow, ultimately leading to long-term sensorimotor recovery in focal ischemic stroke. Jia et al. (2022) demonstrated the role of Salvianic acid A in enhancing angiogenesis *in vivo* and *in vitro* by regulating the PI3K/Akt/mTOR signaling pathway and upregulating VEGF expression.

### 3.3 Anti-inflammatory effects

Severe neuroinflammation during brain ischemia can lead to CIRI, BBB destruction, neuron damage, and vascular aging, further aggravating brain injury (Xu S. et al., 2020). In addition, excessive ROS accumulation can induce inflammation and lead to microcirculation disorders in brain tissue, thus impairing the neurons in the central nervous system (CNS) (Xiang et al., 2021). Research suggests that Salvianic acid A can prevent CIRI through an anti-inflammatory pathway, which involves inhibition of the TLR4–NFκB signal transduction pathway and a decrease in inflammation-associated proteins such as TLR4, NF-κB, NOD-like receptor thermal protein domain associated protein 3, and TNF-α. By decreasing downstream inflammatory factors such as caspase-1, IL-1β, and IL-18, it achieves protection of nerves injured by brain I/R effects (Xv et al., 2018; Yang et al., 2009).

Furthermore, the combination of Salvianic acid A and HSYA has been observed to have stronger anti-inflammatory and antioxidant neuroprotective effects on ischemic tissue, which reflects the unique advantages of compound compatibility in TCM theory (Xu et al., 2017). Owing to its antioxidant and anti-inflammatory properties, Salvianic acid A shows significant protective effects when administered alone; furthermore, the combination of Salvianic acid A and HSYA may have additive or synergistic protective effects, resulting in the enhancement of its neuroprotective effects against CIRI.

### 3.4 Anti-apoptosis

Apoptosis is one of the crucial elements contributing to cerebral I/R (Tuo et al., 2022). Protecting ischemic core cells or penumbra cells from the interference of apoptosis signals is one of the common therapeutic strategies for cerebral I/R injury (Uzdensky, 2019). Salvianic acid A can also regulate some apoptosis signaling pathways in the brain. Several studies have found that intravenous injection of Salvianic acid A can inhibit caspase-3/7 activity by activating the PI3K/Akt signaling pathway to regulate the expression of apoptotic genes Bax and Bcl-2 (Guo et al., 2015), inhibiting the phosphorylation of AKT1 and mTOR by activating AKT1 to reduce the rate of nerve cell apoptosis or improving the neurological dysfunction and pathological morphology in brain tissues of damaged areas, in turn alleviating cerebral ischemia tissue reperfusion injury (Yu et al., 2014; Yu et al., 2015; Zhang et al., 2019).

### 3.5 Inhibition of the BBB and drug penetration

As the most selective physiological barrier in the human body (Liao et al., 2023), the BBB protects the CNS from potentially harmful substances, although this also presents considerable challenges for the treatment of CNS disease (Nilles et al., 2022). Many drugs cannot penetrate the BBB, resulting in an insufficient effective concentration of drugs (Yang et al., 2019). P-glycoprotein is an efflux transporter in the brain capillaries that form the BBB that functions as a crucial factor in the entry of therapeutic drugs to the CNS (Chai et al., 2022). Yu et al. (2011) reported that Salvianic acid A can synergize with cerebral I/R to inhibit P-glycoprotein expression, significantly increasing the solubility of Salvianic acid A in brain tissue, with positive effects on the treatment of ischemic cerebrovascular disease. Nevertheless, more research is necessary to clarify the specific mechanisms of its effects.

Although Salvianic acid A, as a small polar molecule, is difficult to accumulate in large quantities in the brain, its multi-pathway protective effects on CIRI are observable. Researchers have developed a number of Salvianic acid A derivatives and delivery platforms to enhance its ability to cross the BBB (Hui et al., 2018). Interestingly, in previous studies, Salvianic acid A seems to promote new neuron generation in CIRI, which has attracted considerable research interest, and further studies are anticipated (Yu et al., 2005).

## 4 Protective effect of Salvianic acid A against renal I/R injury

With the increasing incidence of hematological malignancies, the frequency of renal I/R injury after kidney resection, kidney transplantation, and complex cardiovascular surgery has increased gradually (Chatauret et al., 2014). Renal I/R injury can also directly or indirectly cause kidney disease, including acute kidney injury, and delayed renal function transplantation, which has adverse postoperative consequences (Saat et al., 2016).

One study found that the anti-inflammatory and antioxidant effects of Salvianic acid A can protect against renal I/R injury to some degree and reduce the occurrence and progression of subsequent kidney disease. Wang reported that Salvianic acid A can reduce MPO activity and inhibit the expression of inflammatory factors such as IL-1β, IL-6, and TNF-α (Wang, 2014). The authors also found that Salvianic acid A increased SOD expression and inhibited MDA expression through anti-inflammatory and antioxidant effects to attenuate the effects of renal I/R injury in mice.

Furthermore, Salvianic acid A can inhibit inflammatory and oxidative reactions in the process of renal injury through the regulation of Nrf2/HO-1, NF-ĸB (Yu et al., 2021), and TGF-β/Smad3 (Guan et al., 2015), and can modulate the expression of factors such as IL-1β, IL-6, TNF-α, MDA, and SOD (Wang, 2014), thus reducing renal injury, particularly renal toxicity induced by chronic renal injury and cisplatin.

As renal I/R injury conditions are increasingly widespread, there is an increased focus on their prevention and treatment, and Salvianic acid A is a good candidate; however, unlike that in the heart and brain, the protective role of Salvianic acid A in renal I/R injury needs to be further validated and researched, and more mechanisms for this role may be unraveled.

## 5 Protective effect of Salvianic acid A against liver I/R injury

I/R injury in the liver is mostly caused during liver transplantation to treat end-stage liver disease (Gazia et al., 2021). Liver function is significantly affected by I/R injury, which in turn affects the prognosis and survival rate of recipients of liver transplants and also limits the success of transplantation (Quesnelle et al., 2015). Salvianic acid A, as a common protective agent against I/R organ damage, exerts its effects through targets in the liver. Huang et al. (2018) reported that Salvianic acid A pretreatment could modulate the Nrf2–HO-1 axis to inhibit p22phox protein expression to counteract oxidative damage, and could increase TGF-β1 expression and downregulate intercellular cell adhesion molecule-1 expression to inhibit the inflammatory response, thus achieving protective effects on liver I/R injury.

Salvianic acid A has also been identified to have favorable protective effects against acute and chronic liver injury that is triggered by a variety of stimuli, including omethoate poisoning (Ren et al., 2010), iron overload (Zhang et al., 2020), carbon tetrachloride (Wang et al., 2007), acetaminophen (Zhou et al., 2015), and alcohol toxicity *in vivo* and *in vitro* (Lan et al., 2020). The protective mechanisms mainly involve antioxidant, anti-inflammatory, and anti-apoptosis effects, along with the maintenance of mitochondrial function and promotion of blood circulation. We postulate that Salvianic acid A exerts a considerable protective effect on liver injury.

Overall, the protective effect of Salvianic acid A on liver I/R injury still has great research potential for the future. Through in-depth research and clinical trials, the mechanism of action and clinical application prospects of Salvianic acid A could be clarified, in addition to providing more effective approaches for the treatment of liver I/R injury.

## 6 Protective effect of Salvianic acid A against intestinal I/R injury

Intestinal I/R injury mostly occurs during major cardiovascular surgery (Mallick et al., 2004). Increased intestinal permeability after cell injury leads to interstitial edema and increased intestinal endotoxin and bacterial translocation, triggering inflammatory responses, oxidative stress, and other injury mechanisms that ultimately aggravate intestinal ischemia injury (Schoenberg and Beger, 1993). The effects of Salvianic acid A on the microcirculatory system make a significant contribution to intestinal I/R injury treatment as well. Research suggests that Salvianic acid A can reduce peroxide production, leukocyte adhesion, and albumin leakage through the mesenteric vein wall for I/R-induced microcirculation dysfunction irrespective of whether Salvianic acid A is administered as a pre-treatment or after the onset of ischemia, while mast cell granulation can only be inhibited by Salvianic acid A before treatment (Han et al., 2009). The results show that Salvianic acid A exerts its therapeutic action on intestinal I/R injury via antioxidant systems and inhibiting CD11b/CD18, a leukocyte adhesion molecule.

Research on applications of Salvianic acid A in the treatment of intestinal I/R injury is still limited, and the relevant studies are still in the primary stages. Additional studies are required to verify its efficacy and provide novel, effective options for the treatment of intestinal I/R injury.

## 7 Protective effect of Salvianic acid A against I/R injury in other organs

Salvianic acid A also exerts protective effects on other organs against I/R damage. In retinal I/R injury, VEGF shows an early increase and a late decrease (Muthusamy et al., 2014). A study found that Salvianic acid A sodium did not significantly affect VEGF expression in the initial stages of reperfusion but downregulates VEGF in the late stages of injury, indicating that Salvianic acid A has a certain preventive effect on retinal neovascularization (Ding et al., 2006). I/R injury of skeletal muscle causes a series of physiological and pathological reactions, affecting normal activities of the body (Delay et al., 2017). The specific underlying mechanism may be that after activation, leukocytes migrate and adhere to the surrounding venous endothelial cells, aggravating ischemic injury and preventing microvascular reperfusion by releasing active factors or damaging the microvascular barrier (Gillani et al., 2012). It has been reported that Salvianic acid A can reduce the levels of creatine kinase, aspartate aminotransferase, lactate dehydrogenase, and MDA; raise SOD activity; and attenuate I/R injury in rabbit hindlimb skeletal muscle through its antioxidant effects (Feng et al., 1990).

Current research on Salvianic acid A in I/R injury has indeed focused mainly on the brain and the heart, with fewer studies focusing on other organs. This may be related to the fact that the brain and heart, as important organs in the human body, are more obviously and severely affected by I/R injury. The anti-inflammatory and antioxidant effects of Salvianic acid A have been verified in various organs. However, understanding the specific role of Salvianic acid A in different organs could optimize its therapeutic application for the treatment of I/R injury in specific organs. In future studies, researchers could apply histological techniques to explore the interactions of multiple signaling pathways associated with Salvianic acid A in depth for a comprehensive understanding of its preventive and therapeutic potential in I/R injury.

## 8 Conclusion

*Salvia miltiorrhiza* has been used in TCM for thousands of years, and its safety and efficacy have been verified for a long period. Salvianic acid A, as one of many water-soluble phenolic acids contained in *S. miltiorrhiza*, has attracted considerable research interest because of its protective effect on many organs. Salvianic acid A has a variety of pharmacologic effects, including anti-oxidation, anti-inflammation, and anti-apoptosis properties; reducing calcium overload; inhibiting platelet aggregation: improving microcirculation; and protecting endothelial cells, and has a protective function in I/R injury in the cardiovascular system, brain, liver, kidneys, and gastrointestinal tract. Researchers have identified numerous relevant signaling pathways and targets that are implicated in the protection of Salvianic acid A during organ damage. Understanding such mechanisms may also facilitate the identification of additional diseases that may respond to Salvianic acid A treatment.

However, the structural characteristics of Salvianic acid A limit its research and application to some extent. The molecular weight of Salvianic acid A is not high; however, the contents of polar groups in the entire structure are relatively high. As a result of this structure, Salvianic acid A has poor lipid solubility and is not able to enter tissues and organs through biofilms after being absorbed and distributed in the body via blood. In particular, the concentration of Salvianic acid A entering the brain is very low due to difficulty in penetrating the BBB, which is a key reason why the therapeutic effect of Salvianic acid A has not met expectations. In this review, we identify many studies investigating how to improve the fat solubility of Salvianic acid A and increase the effective concentration of Salvianic acid A in organs. There are two main approaches. The first is to modify the structure of Salvianic acid A. By modifying the groups, structural polarity can be adjusted to balance lipid solubility and water solubility and improve Salvianic acid A bioavailability. The second approach focuses on drug delivery, new materials, or biotechnology to establish a carrier system for Salvianic acid A, which can deliver Salvianic acid A to the target tissue and release it at a fixed point, thereby increasing the effective concentration at a focal site. The studies above propose novel strategies for overcoming the limitations on the therapeutic effect of Salvianic acid A, and satisfactory results have been obtained in studies at the cellular level and in animal models.

Salvianic acid A has considerable potential in the treatment of I/R injury in various organs. However, there are considerable restrictions on Salvianic acid A research and its application in clinical practice. Future developments in science and technology and the pharmaceutical industry will facilitate further research to overcome the limitations and promote the use of Salvianic acid A against ischemic injury.
