# Supplementary material for: Protective effects of Salvianic acid A against multiple-organ ischemia-reperfusion injury: a review
Source: Front Pharmacol. 2023 Nov 27;14:1297124. doi: 10.3389/fphar.2023.1297124 (PMC10712837; doi:10.3389/fphar.2023.1297124)
Supplement: Supplementary file 1 [file DataSheet3.PDF]

Supplementary Material

1 Supplementary Tables

Supplementary Table 1. Therapeutic and protective mechanisms of Salvianic acid A in different organs after ischemia-reperfusion injury.

Organ	*In vivo/ vitro*	Model	Signaling pathway	Mechanism	Reference
Heart	*In vitro*	Langendorff isolated rat hearts	Sirt1/FoxO1/Rab 7	Anti-oxidative	[60]
	In vivo	ISO-induced myocardial injury rat hearts	Nrf2/HO-1 and PI3K/Akt	Anti-oxidative and anti-apoptotic	[61]
	In vitro	Langendorff isolated rat hearts	Akt/ERK1/2/Nrf2	Anti-oxidative	[62]
	In vivo	ISO-induced myocardial hypertrophy rat hearts	/	Anti-oxidative	[63]
	In vivo	Langendorff isolated rat hearts	/	Anti-oxidative stress and regulation of energy metabolism	[64]
	In vivo	ISO-induced myocardial injury rat hearts	Nrf2	Anti-oxidative and anti-apoptotic	[65]

In vitro	Human umbilical vein endothelial cells	PI3K-Akt	Anti-oxidative and anti-apoptotic	[66]
In vivo	MI injury rat hearts	/	Anti-oxidative	[67]
In vivo and *in vitro*	MI/R injury rat hearts, H9c2 cells	PI3K/Akt and ERK1/2	Anti-apoptotic	[70]
In vivo and *in vitro*	MIRI rat hearts, neonatal SI/R injury cardiomyocytes	RISK	Anti-inflammatory, anti-oxidative, and anti-apoptotic	[71]
In vivo and *in vitro*	MIRI rat hearts, H/R injury NRVM	PAF/PAFR	Anti-apoptotic	[72]
In vivo and *in vitro*	MIRI rat hearts, H9c2 cells	Akt/Nrf2/HO-1	Anti-oxidative and anti-apoptotic	[73]
In vivo	MIRI rat hearts	/	Anti-inflammatory	[76]
In vivo	MI injury rat hearts	/	Reversal of metabolic changes	[77]
In vivo	Langendorff rat hearts	FoxO1-MPTP	Improvement of mitochondrial function	[80]
In vitro	Langendorff rat hearts, H9c2 cells	MPTP	Improvement of mitochondrial function	[81]

	In vitro	H/R injury cardiomyocytes, MIRI rat hearts	mTOR	Anti-apoptosis and anti-autophagy	[84]
	In vitro	H9c2 cells	AMPK-mTOR-Ulk1	Anti-apoptosis and anti-autophagy	[85]
	In vitro	5-HT pre-constriction of coronary arteries	Ca^{2+}	Inhibition of calcium channels	[88]
	In vivo	Guinea pig ventricular myocytes	L-Ca^{2+}	Inhibition of calcium channels	[89]
	In vivo	MI injury rat hearts	L-type calcium current	Inhibition of calcium channels	[90]
	In vitro	H9c2 cells	p-JNK-NF-κB-TRPC6	Anti-apoptosis	[91]
	In vivo	MI injury rat hearts	SDF-1α/CXCR4	Angiogenesis promotion	[95]
Brain	*In vivo*	MCAO rats	/	Anti-oxidative	[100]
	In vivo	CIRI rat brains	/	Anti-oxidative	[103]
	In vivo	Ischemic stroke mice	/	Promotes endogenous neurogenesis	[106]
	In vivo	MCAO/R rat brains	TLR4-NFκB	Anti-inflammatory and anti-apoptotic	[109]

	In vivo	CIRI rat brains	TLR4/NF-κ B and Nrf2/HO-1	Anti-inflammatory and anti-oxidative	[110]
	In vivo	MCAO/R rat brains	NLRP3-caspase-3	Anti-inflammatory	[111]
	In vivo	MCAO/R rat brains	PI3K/Akt	Anti-apoptotic	[114]
	In vivo	MCAO/R rat brains	/	Anti-apoptotic	[115]
	In vivo and *in vitro*	HAPI, PC12 cells, CIRI rat brains	AKT1	Anti-apoptotic	[39]
	In vivo	Verapamil pre-treatment rat brains	/	Inhibition of BBB expression	[120]
Kidney	*In vivo*	Kidney I/R injury rats	/	Anti-inflammatory and anti-oxidative	[123]
	In vivo	Cisplatin damaged kidney	Nrf2/HO-1 and NF-κB	Anti-inflammatory and anti-oxidative	[124]
	In vivo	Chronic kidney injury rat kidneys	NF-κB and TGF-β/Smad3	Anti-inflammatory and anti-apoptotic	[125]
Liver	*In vivo*	Liver I/R injury rats	/	Anti-inflammatory and anti-oxidative	[128]

	In vivo	Acute omethoate poisoning rats	/	Anti-inflammatory	[129]
	In vivo	Iron overload mice	/	Anti-inflammatory, anti-oxidative, and anti-apoptotic	[130]
	In vivo	CCl_4-induced liver injury rats	/	Antioxidant	[131]
	In vivo	APAP-induced liver injury rat primary liver cells	/	Antioxidant	[132]
	In vivo	ALD rats	BRD4/HMGB1	Anti-inflammatory	[133]
Mesentery	*In vivo*	Local MESenteric I/R injury rats	/	Antioxidant	[136]
Retina	*In vivo*	Retina I/R injury rabbit	/	Reduction in VEGF expression	[138]
Limb	*In vivo*	Rabbit right hindlimb I/R injury model	/	Antioxidant	[141]
